# Pain-free survival after endoscopic neurotomy versus radiofrequency ablation of the C2 dorsal root ganglion for cervicogenic headache: a real-world comparison study

**DOI:** 10.3389/fpain.2026.1856069

**Published:** 2026-06-08

**Authors:** Yunjian Huang, Shiyun Xu, Siyi Liu, Wancheng Zheng, Zaiying Jiang, Xiangdong Wan, Xiangyu Zhang, Tao Du, Guang Lu, Bing Ni, Hongwei Zhu

**Affiliations:** 1 Department of Neurosurgery, Xuanwu Hospital Capital Medical University, Beijing, China; 2 Department of Neurology, Xuanwu Hospital Capital Medical University, Beijing, China

**Keywords:** cervicogenic headache, dorsal root ganglion, endoscopic neurotomy, radiofrequency ablation, pain treatment

## Abstract

**Background:**

Cervicogenic headache (CEH) is commonly treated with radiofrequency ablation (RFA) targeting the C2 dorsal root ganglion (DRG), yet the durability of analgesia is often limited. Endoscopic neurotomy (EN) enables direct visualization of the target neural structures and may provide more sustained pain relief; however, supporting evidence remains limited. We compared long-term outcomes of EN versus RFA targeting the C2 DRG in CEH.

**Methods:**

Patients with a positive diagnostic C2 DRG block were treated with EN or RFA. The Numerical Rating Scale (NRS), EuroQol 5-Dimension 5-Level (EQ-5D-5L), and Patient Global Impression of Change (PGIC) were obtained at baseline, and at 3 and 12 months postoperatively. The pain-free duration was recorded at every follow-up. The final follow-up was conducted in January 2026.

**Results:**

Of 76 included patients, 21 underwent EN and 55 underwent RFA. Both groups demonstrated significant reductions in NRS and improvements in EQ-5D-5L at 3 and 12 months postoperatively compared with baseline (*P* < 0.001). NRS and EQ-5D-5L outcomes were comparable between groups at 3 months (*P* > 0.05), whereas EN showed superior effectiveness at 12 months (*P* < 0.05). PGIC favored EN at both 3 and 12 months (*P* < 0.05). No serious adverse events were observed in either group, although occipital-distribution numbness occurred in 21/21 (100%) EN patients and 48/55 (87.3%) RFA patients. Median pain-free duration was 33 months in the EN group and 8 months in the RFA group (*P* < 0.05).

**Conclusions:**

In this retrospective cohort, both EN and RFA improved pain and quality of life in clinically selected patients with CEH. EN was associated with longer pain-free duration; however, given its invasiveness and the diagnostic overlap between CEH and occipital neuralgia, EN should be considered cautiously as an escalation option in carefully evaluated patients.

## Introduction

Cervicogenic headache (CEH) is a secondary headache attributed to disorders of the upper cervical spine and/or its soft tissues, in which head pain is referred from a nociceptive source in the neck (1, 2). CEH is frequently accompanied by neck pain, restricted cervical range of motion, functional limitations, and impaired quality of life, with an estimated prevalence of up to 4% in the general population (3, 4). Although CEH is a common type of headache, pharmacotherapy and physical therapy often have limited efficacy and frequently fail to provide long-term relief.

Pathophysiologically, CEH may result from the convergence of nociceptive afferent input from the upper cervical spinal nerves within the trigeminocervical complex. The C2 DRG may be particularly relevant because of its relatively large size and proximity to C1–2 osteophytes, venous congestion, and vertebral artery pulsation, which together provide a biological rationale for C2 DRG-targeted interventions (5). Consequently, image-guided RFA and pulsed radiofrequency (PRF) targeting the C2 DRG are widely used in clinical practice. However, the clinical benefit of conventional RFA/PRF for CEH is often short-lived, and repeat procedures may be required when symptoms recur, underscoring the need for more durable techniques (6, 7).

Over the past two decades, minimally invasive spine surgery has evolved to provide magnified visualization of fine anatomic structures in an aqueous environment. This capability enables direct identification of target spinal/neural elements and facilitates neurotomy under endoscopic guidance, which may reduce the risk of incomplete lesioning and subsequent recurrence. Nevertheless, evidence supporting endoscopic neurotomy (EN) for CEH has been largely limited to case reports (8), and comparative data against RFA—particularly regarding pain-free survival—remain scarce. To address this evidence gap, we conducted a real-world comparison study that used the Patient Global Impression of Change (PGIC) to compare pain-free survival after EN and RFA, and to further assess the effects of these treatments on pain relief and quality of life in patients with CEH.

## Materials and methods

### Study design

The study was approved by the Ethics Committee of Xuanwu Hospital, Capital Medical University (KS2024140), and written informed consent was obtained from all participants or their legal representatives.

**Figure 1:**
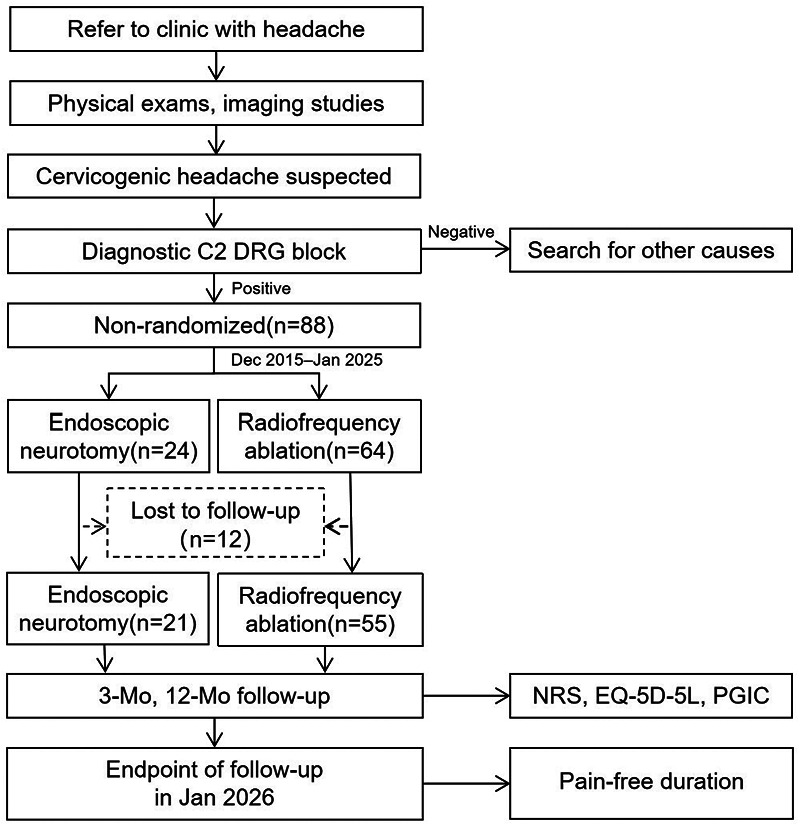
Study flowchart. DRG, dorsal root ganglion; NRS, Numerical Rating Scale; EQ-5D-5L, EuroQol 5-Dimension 5-Level Questionnaire; PGIC, Patient Global Impression of Change.

Patients were consecutively enrolled at a single medical center between December 2015 and January 2025. The inclusion criteria were: (1) age between 20 and 80 years; (2) diagnosis of CEH according to the third edition of the International Classification of Headache Disorders (9); (3) pain duration ≥ 6 months with inadequate response to conventional treatment, including medication, physiotherapy, and lifestyle modification; (4) a Numerical Rating Scale (NRS) ≥ 5 at baseline; and (5) a positive response to diagnostic C2 DRG block. At our center, CEH was diagnosed based on an integrated clinical evaluation rather than diagnostic block alone, including headache features and cervical findings suggestive of CEH, such as unilateral headache with neck pain, aggravation by neck movement or sustained awkward posture, restricted cervical motion, and/or upper cervical tenderness. Patients with features more suggestive of occipital neuralgia, including paroxysmal stabbing or shooting pain in the distribution of the greater or lesser occipital nerves, marked occipital nerve tenderness or allodynia, or evidence of peripheral occipital nerve entrapment, were excluded. Before intervention, all patients underwent detailed history taking, neurological examination, and cervical imaging review to exclude other structural causes of headache and support the diagnosis of CEH. Exclusion criteria were: (1) symptomatic cervical spondylosis; (2) untreated coagulopathy; (3) inability to complete the rating scales; (4) cognitive dysfunction; (5) psychiatric illness; and (6) follow-up duration < 12 months. Patients were divided into two groups according to the treatment strategy: EN and RFA. In our real-world practice, because EN is more invasive than percutaneous RFA, it was generally considered only after failure of conservative treatment and usually in patients with recurrent pain or inadequate durability after previous RFA. Conversely, RFA was suggested as the initial interventional treatment (Figure 1).

### Procedure

#### Endoscopic neurotomy

**Figure 2:**
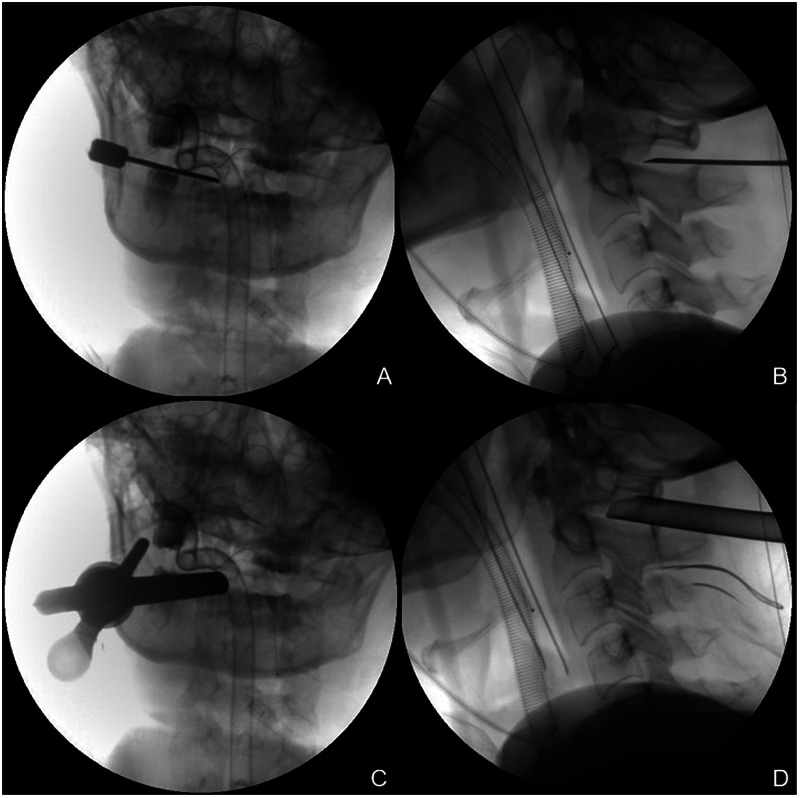
Ideal positions of the puncture needle [**(A)** A-P view; **(B)** LAT view] and the working cannula [**(C)** A-P view; **(D)** LAT view] for the C2 dorsal root ganglion. A-P, anteroposterior; LAT, lateral.

After induction of general anesthesia, the patient was positioned prone with the neck maintained in a neutral alignment. A C-arm fluoroscope was used to identify the skin projection point of mid C1–2 atlantoaxial joint on the affected side. The skin entry point was selected 0.5 cm medial and 1 cm caudal to the skin projection point. The puncture needle with stylet was directed toward the C1–2 atlantoaxial joint. The needle tip was adjusted under anteroposterior (A-P) and lateral (LAT) fluoroscopy until it was located at the midpoint of the C1–2 atlantoaxial joint on the A-P view and posterior to the joint on the LAT view. The guidewire was then exchanged. A 1 cm incision was made through the skin and subcutaneous tissue, followed by soft-tissue dilation and insertion of the working cannula. Correct positioning of the cannula was reconfirmed using A-P and LAT views (Figure 2).

**Figure 3:**
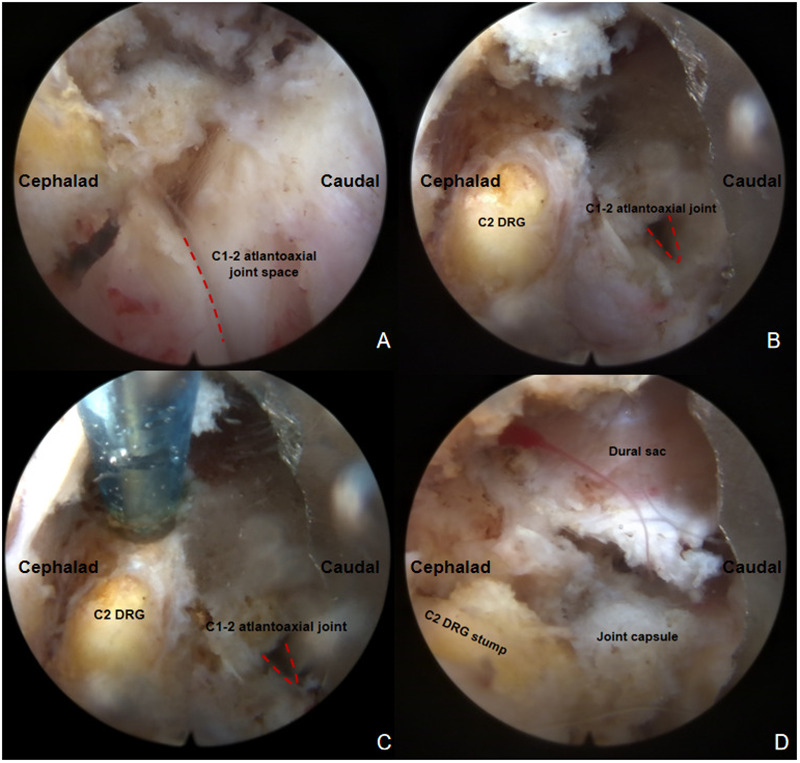
Endoscopic views. The lateral C1–2 atlantoaxial joint space was identified **(A).** Further cephalad exploration revealed a markedly thickened, yellowish C2 DRG **(B)**, which was transected under endoscopic guidance **(C)**, with the C2 DRG stump subsequently observed **(D).** DRG, dorsal root ganglion.

Under endoscopic visualization, a bipolar radiofrequency electrode was used to remove soft tissue, thereby exposing the superior border of C2 lamina and the ligamentum flavum. Following fenestration of the ligamentum flavum, the dural sac was exposed. The lateral aspect of the dural sac was subsequently explored. Prior to exploration of the lateral aspect of the dural sac, prophylactic hemostasis was achieved using hemostatic gauze. Exploration was continued along the superior border of C2 lamina toward the C1–2 atlantoaxial joint, where the joint space was identified. Further cephalad exploration revealed a markedly thickened, yellowish C2 DRG above the C1 lateral mass, which was transected under endoscopic guidance. After removal of the prophylactic hemostatic gauze, the working cannula was withdrawn, and the incision was closed using an intradermal suture (Figure 3).

#### Radiofrequency ablation

In patients undergoing RFA, the procedure was performed with the patient in the prone position under local anesthesia. A puncture needle was advanced to the C2 DRG under A-P and LAT fluoroscopy according to the positions described above. Sensory stimulation (100 Hz, 200 μs pulse width) was performed and elicited paresthesia concordant with the patient's pain distribution at < 1 V. Motor stimulation (2 Hz) produced visible or palpable contractions of the suboccipital and upper posterior cervical muscles at < 1 V. Thermal RFA was then performed at 80 °C for 120 s; the electrode was withdrawn by 1 mm and the lesion was repeated.

#### Clinical evaluation

Clinical evaluation was administered by an independent interviewer blinded to the patients' treatment assignments. Demographic data and procedure characteristics, including age, sex, pain duration, fluoroscopy time and operative time were collected. Baseline NRS (10) and utility score of EuroQol 5-Dimension 5-Level questionnaire (EQ-5D-5L) (11, 12) were obtained before the diagnostic block. During the follow-up, NRS, utility score, and Patient Global Impression of Change (PGIC) were obtained at 3 and 12 months after the procedure. PGIC was assessed on a 7-point scale ranging from 1 to 7 (1 = very much improved; 2 = moderately improved; 3 = slightly improved; 4 = no change; 5 = slightly worsened; 6 = moderately worsened; 7 = very much worsened). If a patient underwent reoperation because of recurrence of pain within the 3- or 12-month follow-up, the surveys were obtained before the reoperation and were accounted for in 3- and 12-month follow-up, respectively. Pain-free survival was defined as the time from the procedure to pain recurrence, with “pain-free” operationalized as a PGIC rating of 1–2. The final follow-up was conducted in January 2026 (Figure 1).

#### Statistical analysis

Analyses were performed using IBM SPSS 27 (IBM Corp., New York, NY, USA). A two-sided *P* value < 0.05 was considered statistically significant. Normally distributed continuous variables were reported as mean ± SD and were compared using an independent *t*-test, whereas skewed variables were reported as median (*P25–P75*) and were compared using the Mann–Whitney *U* test. Categorical data were presented as n (%) and were compared using Pearson's chi-square test or Yates' corrected chi-square test. Within-group changes were assessed using the Wilcoxon signed-rank test. Kaplan–Meier analysis was used for comparison of pain-free survival between groups, and the difference was analyzed by the log-rank test.

## Results

Of the 88 enrolled patients, 12 were lost to follow-up. Ultimately, 21 patients in the EN group and 55 in the RFA group were included in the final analysis.

Descriptive statistics for demographic and procedural characteristics are summarized in Table 1. Compared with RFA, EN was associated with significantly longer fluoroscopy time and operative time (*P* < 0.001).

**Table 1:** Demographic data and procedure characteristics.

Characteristics	Endoscopic neurotomy (*n* = 21)	Radiofrequency ablation (*n* = 55)	Overall (*n* = 76)	*P* value
Demographic data				
Age (years)	50.9 ± 11.0	51.7 ± 11.8	51.5 ± 11.5	0.780
Sex				0.460^a^
Male	11 (52.4)	24 (43.6)	35 (46.1)	
Female	10 (47.6)	31 (56.4)	41 (53.9)	
Pain duration (years)	11 (8.5,16)	6 (1,20)	8.5 (2.25,18.25)	0.077^b^
Follow-up duration (months)	24 (16.5,30)	17 (14,30)	19.5 (14,29.75)	0.272^b^
Procedure characteristics				
Laterality				0.374^c^
Unilateral	18 (85.7)	40 (72.7)	58 (76.3)	
Bilateral	3 (14.3)	15 (27.3)	18 (23.7)	
Fluoroscopy time (s)	24.3 ± 7.3	16.9 ± 5.8	19.0 ± 7.0	**<0**.**001**
Operative time (min)	68.8 ± 8.2	28.6 ± 8.1	39.7 ± 19.8	**<0**.**001**

Data are shown as mean ± SD, median (*P25, P75*) or *n* (%); Statistical analyses were performed using independent t-tests unless a-c.

a From Pearson's *chi*-squared test.

b From Mann–Whitney U test.

c From Yates’ corrected chi-square test.

Bold *P* values indicate statistically significant differences between groups (*P* < 0.05).

No serious adverse events, including infection, hemorrhage, spinal cord injury, or upper limb dysfunction, were reported in either group. Occipital-distribution numbness of varying severity occurred in 21/21 (100%) patients in the EN group and 48/55 (87.3%) patients in the RFA group. Table 2 presents changes in NRS, utility score, and PGIC over the 12-month follow-up period. In both groups, NRS scores significantly decreased and utility scores significantly increased at 3 and 12 months compared with baseline (*P* < 0.001). Based on the NRS and utility scores, EN showed comparable effectiveness to RFA at 3 months (*P* > 0.05). However, at 12 months, EN was significantly more effective than RFA (*P* < 0.05). PGIC scores indicated greater clinical improvement in the EN group than in the RFA group at both 3 months and 12 months (*P* < 0.05).

**Table 2:** Baseline and postoperative NRS, utility score, and PGIC.

Outcome measures	Endoscopic neurotomy (*n* = 21)	Radiofrequency ablation (*n* = 55)	Overall (*n* = 76)	*P* value
NRS				
Baseline	7 (6,8.5)	8 (7,8)	7 (6,8)	0.730
3 months	2 (0.5,3)*	4 (0,7)*	3 (0,6)	0.082
12 months	3 (1,4)*	5 (2,7)*	4 (2,7)	**0** **.** **035**
Utility score				
Baseline	0.54 (0.40,0.70)	0.54 (0.54,0.54)	0.54 (0.54,0.70)	0.789
3 months	0.94 (0.94,0.97)*	0.70 (0.54,1.00)*	0.94 (0.70,1.00)	0.073
12 months	0.94 (0.70,0.97)*	0.70 (0.54,0.94)*	0.70 (0.54,0.94)	**0** **.** **032**
PGIC				
3 months	1 (1,2)	2 (1,4)	2 (1,4)	**0** **.** **008**
12 months	1 (1,2.5)	4 (1,4)	2 (1,4)	**0** **.** **019**

Data are shown as median (*P25, P75*); *Compared with baseline, the change was significant (*P* < 0.001); Statistical analysis was performed using the Mann–Whitney U test; NRS, Numerical Rating Scale; PGIC, Patient Global Impression of Change.

Bold *P* values indicate statistically significant differences between groups (*P* < 0.05).

**Figure 4:**
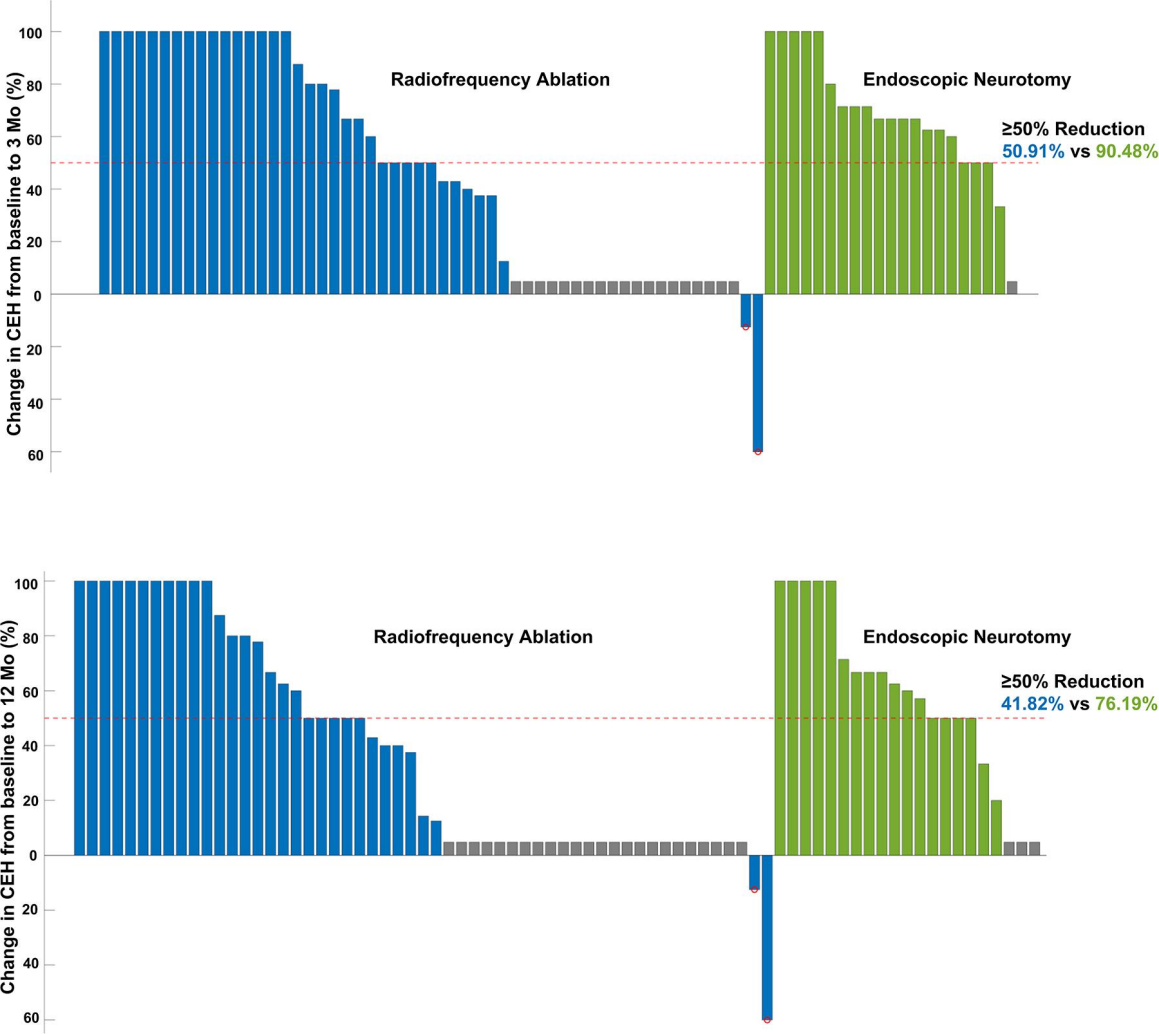
Waterfall plot of individual percentage changes in NRS from baseline at 3 and 12 months. A ≥ 50% reduction in NRS was achieved in 90.48% (EN) vs. 50.91% (RFA) at 3 months and in 76.19% vs. 41.82% at 12 months.

Figure 4 shows individual NRS responses at 3 and 12 months after the intervention, expressed as percentage change from baseline. A ≥ 50% reduction in NRS was achieved in 90.48% of patients in the EN group and 50.91% in the RFA group at 3 months, and in 76.19% and 41.82% of patients, respectively, at 12 months.

**Figure 5:**
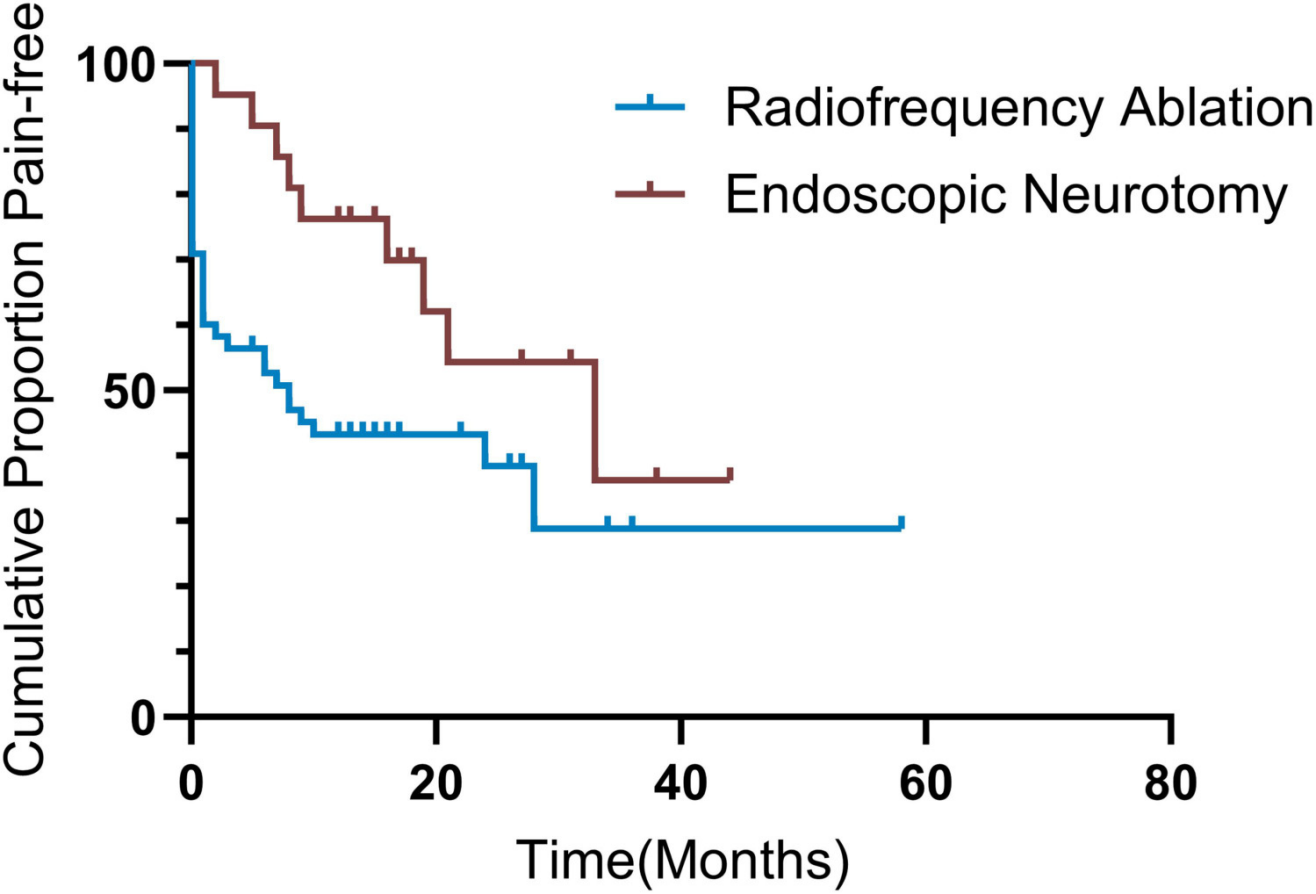
Kaplan-Meier curves for pain-free survival after endoscopic neurotomy and radiofrequency ablation. Median pain-free duration was 33 months and 8 months, respectively (*P* < 0.05).

With a follow-up duration of 19.5 (14, 29.75) months, Kaplan–Meier analysis demonstrated pain-free survival in the EN and RFA groups (Figure 5). EN was associated with a more durable treatment effect than RFA, with a median pain-free duration of 33 months versus 8 months.

## Discussion

In the retrospective cohort of 76 patients with CEH, both EN and RFA targeting the C2 DRG significantly improved pain intensity and quality of life. Notably, EN was associated with a more durable treatment effect than RFA, with a median pain-free duration of 33 months versus 8 months.

This finding aligns with the broader literature indicating that conventional RFA/PRF can provide meaningful symptom relief in selected patients, but the benefit is often short-lived; moreover, high-quality comparative trials in CEH remain scarce. Recent evidence continues to support image-guided C2-targeting strategies. For example, CT-guided C2 DRG thermal RFA in carefully selected patients has been reported to reduce pain substantially, with generally low rates of major complications, although sensory symptoms such as occipital paresthesia can occur (13). In addition, ultrasound-guided C2 DRG PRF has been reported as feasible with low severe adverse-event rates in retrospective cohorts, highlighting the potential value of improved visualization of adjacent vascular and neural structures (14). However, incomplete lesioning may occur because radiofrequency procedures are not performed under direct visualization.

From an evidence hierarchy perspective, even in related cervical facet–mediated pain conditions, contemporary systematic reviews/meta-analyses suggest moderate evidence supporting cervical radiofrequency neurotomy for chronic neck pain but only limited-to-fair evidence for CEH specifically, emphasizing the need for better-designed CEH trials (15). Therefore, our findings should be interpreted as hypothesis-generating and supportive of further prospective validation rather than definitive superiority.

From a pathophysiological standpoint, nociceptive afferents from the upper cervical segments converge with the trigeminal system within the trigeminocervical complex, allowing pain originating from cervical structures to be referred to the occipital, frontal, orbital, and parietal regions (16). In particular, the C2 DRG and adjacent C2–3 related neural pathways (e.g., greater/lesser occipital nerves and third occipital nerve) are key relay points for referred head pain, providing a strong anatomic rationale for targeting C2 DRG in interventional treatment (16).

RFA and PRF are believed to provide analgesia mainly through interruption or modulation of nociceptive transmission at C2 DRG (17). However, recurrence after RFA/PRF is frequently attributed to nerve regeneration or incomplete lesioning, which can limit durability. EN may improve durability because endoscopic magnification enables direct visualization and more complete neurotomy of C2 DRG, potentially reducing the likelihood of insufficient lesioning and subsequent regeneration.

Based on our inclusion criteria and real-world treatment allocation, EN should not be interpreted as a first-line intervention for all patients with CEH. Rather, given its greater invasiveness, EN may be most appropriate as an escalation strategy for carefully selected patients who (1) fulfill clinical diagnostic criteria for CEH, (2) have failed adequate conservative management, (3) demonstrate supportive response to diagnostic C2 DRG block, and especially (4) experience inadequate or short-lived benefit after prior RFA. In this context, EN may represent an escalation interventional option rather than a routine primary procedure. In both the EN and RFA groups, the most common adverse effect was occipital-distribution numbness, and no patients reported regret attributable to this symptom. No serious adverse events were observed in either group, suggesting an acceptable safety profile in this small cohort with appropriate technique and patient selection. Nevertheless, EN required significantly longer fluoroscopy and operative times than RFA, likely reflecting greater procedural complexity and a steeper learning curve, and may lead to higher resource utilization. Potential risks include intra-arterial or intrathecal puncture and injection of medication, cerebrospinal fluid leak, spinal cord puncture, and vertebral artery injury with a risk of stroke (18).

This study has three limitations that should be acknowledged. First, because this was a retrospective, real-world observational cohort study, treatment allocation was not randomized and may have been influenced by prior treatment response and operator preference, introducing potential selection bias. Second, although patients were diagnosed clinically as having CEH and those with symptoms more suggestive of occipital neuralgia were excluded as much as possible, the overlap between CEH and occipital neuralgia in routine practice means that some degree of diagnostic misclassification cannot be completely ruled out, particularly because a positive C2 DRG block is not specific for CEH. Third, the single-center design and modest sample size may have limited statistical power and generalizability.

## Conclusions

Both EN and RFA targeting the C2 DRG provided clinically meaningful improvements in pain and quality of life in patients diagnosed with CEH in this real-world cohort. EN was associated with longer pain-free survival; however, given its greater invasiveness, it may be best considered an escalation treatment option for carefully selected patients after thorough differential diagnostic evaluation and inadequate or short-lived response to RFA.

## Data Availability

The raw data supporting the conclusions of this article will be made available by the authors, without undue reservation.
